# The Temporally-Integrated Causality Landscape: Reconciling Neuroscientific Theories With the Phenomenology of Consciousness

**DOI:** 10.3389/fnhum.2021.768459

**Published:** 2021-11-04

**Authors:** Jesse J. Winters

**Affiliations:** Department of Psychiatry and Behavioral Sciences, Texas A&M University, College Station, United States

**Keywords:** consciousness, TICL, phenomenology, EM field, causality

## Abstract

In recent years, there has been a proliferation of neuroscientific theories of consciousness. These include theories which explicitly point to EM fields, notably Operational Architectonics and, more recently, the General Resonance Theory. In phenomenological terms, human consciousness is a unified composition of contents. These contents are specific and meaningful, and they exist from a subjective point of view. Human conscious experience is temporally continuous, limited in content, and coherent. Based upon those phenomenal observations, pre-existing theories of consciousness, and a large body of experimental evidence, I derived the Temporally-Integrated Causality Landscape (TICL). In brief, the TICL proposes that the neural correlate of consciousness is a structure of temporally integrated causality occurring over a large portion of the thalamocortical system. This structure is composed of a large, integrated set of neuronal elements (the System), which contains some subsystems, defined as having a higher level of temporally-integrated causality than the System as a whole. Each Subsystem exists from the point of view of the System, in the form of meaningful content. In this article, I review the TICL and consider the importance of EM forces as a mechanism of neural causality. I compare the fundamentals of TICL to those of several other neuroscientific theories. Using five major characteristics of phenomenal consciousness as a standard, I compare the basic tenets of Integrated Information Theory, Global Neuronal Workspace, General Resonance Theory, Operational Architectonics, and the Temporo-spatial Theory of Consciousness with the framework of the TICL. While the literature concerned with these theories tends to focus on different lines of evidence, there are fundamental areas of agreement. This means that, in time, it may be possible for many of them to converge upon the truth. In this analysis, I conclude that a primary distinction which divides these theories is the feature of spatial and temporal nesting. Interestingly, this distinction does not separate along the fault line between theories explicitly concerned with EM fields and those which are not. I believe that reconciliation is possible, at least in principle, among those theories that recognize the following: just as the contents of consciousness are distinctions within consciousness, the neural correlates of conscious content should be distinguishable from but fall within the spatial and temporal boundaries of the full neural correlates of consciousness.

## Introduction

Consciousness, the subjectivity which manifests in the waking and dreaming brain, is perhaps the greatest mystery in all of science. The scientific method is a system for establishing objective matters of fact by the empirical means of observation and controlled experiment. Up to this point, we have no objective means for establishing the existence or lack of existence of a state of consciousness in any system, the human brain or otherwise. As individual human minds, the contents of experience are self-evident and undeniable. It is within our human minds that scientific experiments and explananda have been contrived. We, the conscious minds of human beings, make observations, derive predictions, test hypotheses, and draw conclusions. It is no simple feat to turn the focus from the materials and forces of the objective world, of which we can make such observations, back upon the observer. Consciousness is a subjective experience itself, and only within such an experience can any observation be made. The challenge faced by neuroscientific theorists in this domain is to establish, upon solid evidence, a relationship between conscious events and objective, physical structures or processes in the brain. Toward that aim, a large and growing body of neuroscience literature is making progress. This includes progress in the elucidation of two major theoretical frameworks: Integrated Information Theory (IIT) and Global Neuronal Workspace Theory (GNW). But, serious attention should be extended to include other established frameworks, such as Operational-Architectonics (O-A) and Temporo-Spatial Theory of Consciousness (TTC). In particular, O-A has been making substantial theoretical progress (Fingelkurts et al., 2019, 2020). Important recent efforts have been made to compare and contrast a wide range of consciousness theories (Northoff and Lamme, 2020). Georg Northoff and his colleagues have been working to establish a new direction in cognitive neuroscience with a focus on spatial-temporal dynamics of brain activity (Northoff et al., 2020). It is my opinion that such a project is of great value for advancing our understanding of consciousness and cognition. Here, I extend the process of bringing the Temporally-Integrated Causality Landscape (TICL) into contact with the larger field. The first article on the TICL focused on a contrast with IIT and GNW (Winters, 2020). Beginning from an exploration of phenomenal human consciousness and its contents, I proposed the TICL as a framework for the full neural correlate of consciousness. Here, I revisit the TICL and the phenomenal features of human consciousness upon which it is based. I expand upon the TICL, by making its grounding in physics more explicit and by expanding upon its implications. Subsequently, I compare the insights of the TICL with a wider scope of established neuroscientific frameworks, including some of those (O-A and General Resonance Theory, GRT) which explicitly invoke electromagnetics (EM). A new consolidation among theoretical frameworks, upon the grounds of reason and evidence, might accelerate the progress toward a true understanding of consciousness as a physical phenomenon in the universe. With that scientific understanding in hand, we will be able to establish, with the force of scientific certainty, the subjectivity or lack of subjectivity inhering in a human brain state or that of any other physical system.

### The Temporally-Integrated Causality Landscape

**Figure 1:**
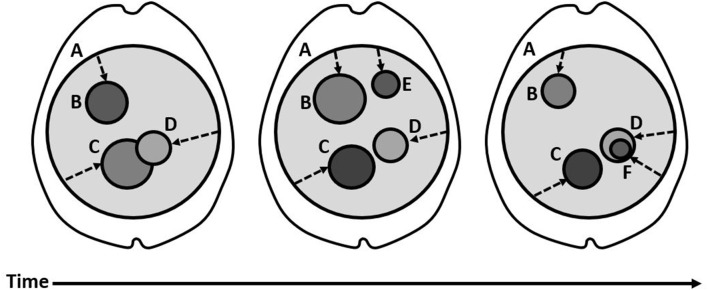
*The Structure of the Temporally-Integrated Causality Landscape*. (A) The large, light gray circle corresponds to the System, which has a non-zero level of TIC and covers a substantial area of the thalamus and cortex. Over time, the System persists with little overall change. The dotted arrows are meant to show that the System is the point of view upon the nested Subsystems. (B–F) The smaller, darker gray circles correspond to Subsystems within the System. (B) This Subsystem shows a change in size and TIC (darkness of color) over time, with less TIC and spatial extent in the third panel relative to the first. (C) This Subsystem shows a change in size and TIC over time, with higher TIC but smaller spatial extent in the third panel than in the first. (D) This Subsystem shows some drift in position relative to Subsystem (C), over the three panels. In the third panel, it comes to contain a smaller Subsystem (F), which has even higher TIC (darker gray). (E) This Subsystem appears briefly in the second panel, then disappears again. F, This small Subsystem appears within the spatial domain of Subsystem D. It has a higher level of TIC (darker gray).

According to the TICL, a distinction can be made between consciousness as a unified whole and the individual contents which compose the unity. Thus, a distinction is made between the System and its Subsystems, which are understood to be the full neural correlates of consciousness and the content-specific neural correlates of consciousness, respectively. Figure 1 illustrates the basic structure of the TICL, with the System (A) represented by a large, light gray circle occupying much of the cortex and the thalamus. The System is that component of the thalamocortical brain which exhibits some non-zero level of temporally-integrated causality (TIC) among all its neuronal elements. In Figure 1, the level of TIC is shown with the darkness of the color gray. The System (A) contains the Subsystems (B-F) which change over time. Figure 1 should be understood as an illustration of concepts. The Subsystems (B-F) do not reflect anatomical accuracy. Integration, in the context of TICL, refers to causal influence in both directions. Thus, integrated elements are characterized by having causality upon one another over some timeframe and, therefore, indirect causality upon their own future state. Among a group of integrated elements, the TIC is the amount of causal influence over the time that it takes to achieve it. The System (A) is irreducible in the sense that it only includes those neuronal elements which are contributing causality and are subject to effects under the influence of the other neuronal elements over some period of time. The System alone is insufficient for consciousness. Evidence of this is provided by global synchrony as might occur with certain types of epileptic seizures which co-occur with loss of conscious experience (Blumenfeld, 2011). The TICL explains this by necessitating the existence of Subsystems (B-F) within the System for the consciousness of content. According to the TICL, a Subsystem is a group of neuronal elements within the System which have a higher level of TIC than the System-at-large, shown in Figure 1 having darker gray colors. This can occur by alteration of the numerator (the amount of causality) or the denominator (the time required) or both. In this way, the activity corresponding to the Subsystem is nested within the time and space of the System. Accordingly, the content which is produced by a Subsystem is nested within the phenomenal time and space produced across the System. The dynamics of Subsystems provide meaningful content from the point of view (the dotted arrows) of the System. These dynamics are illustrated in the figure as changes in the size and grayness of the nested circles (B-F). For example, Subsystem B can be seen to change in size and TIC (grayness), across time. Subsystem E appears only briefly. Subsystem F appears within the pre-existing Subsystem D. The dotted arrows are a crude illustration showing that the System (A) is the point of view upon the Subsystems. Since the Subsystems have higher TIC than the System, they are experienced in specific and meaningful ways. The Subsystemic TIC is intrinsic to the Systemic TIC. In fact, the TICL predicts that the Subsystemic activity is experienced in the form of its geometrical relationship to the System, in space and time. It is thus directly experienced as relational meaning (color, shape, size, pitch, tone, good, bad, painful, strange, scary, sad, interesting, and so on). All neuronal activities in the thalamocortical system which do not contribute to sufficiently high TIC to participate in a Subsystem, are subconscious, background activities. This means that a threshold for consciousness is built-in to the functional organization of the thalamocortical brain.

The brain is a material structure composed of interconnected neurons. Subcellular components, such as axons, dendrites, and pre- and post-synaptic specializations are subject to local causal influences. An important clarification is needed in order to advance the scientific search for consciousness in terms of fundamental physics: Causality requires force. We tend to discuss neuronal function in terms of the movement of charged particles and the interactions of molecules. For example, we understand that sodium, potassium, and chloride ions, moving across the cell membrane, are responsible for the polarization and depolarization of the cell. We know that ion movement across the lipid bilayer requires protein channels, such as voltage-gated sodium channels. However, less discussed in the context of neuronal function is the fundamental force by means of which ions make a difference, namely, the electromagnetic (EM) force (Lorentz Force). There are four fundamental forces in physics: the strong force and the weak force which govern interactions within the atom, the gravitational force, and EM. Ions, biomolecules, and other material substances (composed of atoms) exhibit direct causality upon one another by means of EM forces. Thus, the TICL suggests that the temporally-integrated causality of the System and its Subsystems refers to a complex arrangement of electromagnetic fields. Care should be taken to avoid a naïve conception of nested EM fields in the brain. The thalamocortical brain is a complex system, and human phenomenal consciousness is complex as well. Ultimately, we should expect simple, fundamental principles to underlie consciousness as a phenomenon in the universe. Thus, the study of human consciousness by experimentation poses challenges for deriving those principles. The brain is essentially an EM field system, so the physical measurements we make will inevitably involve the interactions of the EM field. This is the same for all neuroscientific theories. However TIC becomes formulated, in terms of fundamental physics, its empirical verification will be achieved through measurements of EM field properties at a spatial and temporal resolution appropriate to the System and its Subsystems. It would be a mistake to overcommit to a physical formalization of the TICL too early in its theoretical development. Without a doubt, this presents a limitation for distinguishing among frameworks, by experimentation. But, the purpose of this work is not to describe the winning theory in a competition. The purpose is to advance our theoretical understanding of consciousness and to be positively influential in the collaborative process of discovery.

The TICL framework assumes that consciousness of contents is an emergent property of a sufficiently complex system; consciousness of contents requires a unified structure of causality with differentiated structures of causality contained within it. The TICL suggests that a unified (and sustained) thalamocortical EM field structure must co-exist with nested and dynamic Subsystemic EM field structures in order for consciousness to emerge. In contrast to IIT, integration among components of a structure is not predicted to correlate with consciousness, unless there are subsets of components with higher levels of integration to be appreciated by the wider structure. Thus, a physically-nested arrangement is necessary, according to the TICL, for the consciousness of contents. In IIT, consciousness is assumed to be intrinsic to, and limited to, that portion of thalamocortical activity which exhibits maximum causal integration over a discrete timeframe. According to the TICL, there is a distinction between an overarching thalamocortical structure, necessary for consciousness, and embedded thalamocortical structures which are necessary for conscious contents. In IIT, the portion of thalamocortical activity which correlates to consciousness, exists to itself, intrinsically. According to the TICL, the portion of thalamocortical activity which corresponds to conscious content (the Subsystems), exists from the point of view of a larger portion of thalamocortical activity (the System). Subsystemic activities are intrinsic to and meaningful within the System (Winters, 2020). In physical terms, the EM fields which compose Subsystemic structure of causality are nested, in space and time, within the EM field complex which composes the Systemic structure of causality. While this description is reminiscent of dualism, the TICL is explicitly monistic; the contents of consciousness are parts of, or disturbances in, the conscious mind. In an arrangement such as the waking thalamocortical brain, a very large quantity of EM field interactions can occur within the boundaries of the System. The TICL assumes that the integration of causality in time is the key to consciousness. This understanding of causality is not limited to or specific to EM forces. It may be that EM forces are those that are relevant to consciousness as exhibited by the brain, but, in principle, any structure of causality exhibiting both a System and Subsystems could be conscious. The TICL is an attempt to account for consciousness as emergent from the human brain, but it does not rule out consciousness in other systems instantiating the same principles.

### The Phenomenology of Human Consciousness

A robust theory of human consciousness should provide an explanation for the self-evident characteristics of human experience. Again, Nagel defined being conscious as “there is something that it is like to be” (Nagel, 1974). So, what is it like? Phenomenologically, human consciousness is: (1) unified and compositional; its contents are (2) specific and meaningful; and (3) they exist from a subjective point of view. Human consciousness is (4) temporally continuous; and (5) limited and coherent.

#### Consciousness Is a Unified Composition of Contents

First, consciousness is a unified composition of contents (Koch, 2004). Human consciousness always has content. This is what distinguishes conscious states from nonconscious ones. Even if one is totally confused, the particular quality of that confusion of thoughts or sensations is content. Any given experience contains lots of different identifiable contents, such as a visual scene composed of objects arranged in space, sounds and smells, thoughts and feelings. From a subjective point of view, all of these occur in a common, unified experience. We know that auditory and visual stimuli, language comprehension, and feelings of pressure or vibration on the skin, are all processed by different networks in the cerebral cortex. Moreover, the sense of self can be disrupted or made absent pharmacologically, while preserving a unified composition of contents (Millière et al., 2018; Fingelkurts et al., 2020). The neural correlates of conscious unity are thought to involve functional integration, synchrony, or rapid communication in the thalamocortical system, encompassing a range of cortical regions involved in specialized areas of sensory and cognitive conscious contents.

#### Conscious Contents Are Specific and Meaningful

Second, conscious contents are specific and meaningful (Koch, 2004; Koch et al., 2016). A certain sound is different from another sound. A certain thought or feeling is different from any other thought or feeling. Green is different from blue, etc. In the scheme of conscious contents, some things are more alike than others. When we say that consciousness is the fact that “it is like something”, we are referring to the specific and meaningful contents of experience (Nagel, 1974). Since individual contents are distinguishable and, at least in principle, describable, a complete theory of human consciousness must explain the differentiation among structures or processes that makes this possible. The neural correlates of specific conscious contents are thought to involve differentiation of thalamocortical network functions, occurring during conscious states. These differentiated, modular activities should be nested within the spatial boundaries of the full neural correlate of consciousness.

#### Conscious Contents Exist From a Subjective Point of View

Third, conscious contents exist from a point of view. This is subjectivity. Whatever content is being experienced, it is only being experienced within that conscious entity, from that point of view. If there is no point of view to observe content, then there is no consciousness. This is directly related to unity. If no common structure or process integrates the contents, then there is no common point of view, and thus, no conscious entity. From the point of view of the conscious subject, contents exist and have meaning. Interestingly, even absent a concept of self, the point of view is implied by the existence of content experiences under high-dose psychedelics (Millière et al., 2018; Fingelkurts et al., 2020). This is also true in the case of illusions involving disembodiment or autoscopy (Blanke and Metzinger, 2009). The point of view should, therefore, not be confused with self-consciousness.

The subjective nature of consciousness is currently inexplicable to experimentation, and thus requires a philosophical consideration in addition to an empirical one. This is in evidence in the “Unfolding Argument”, which makes the case that any recurrent computation (input-output relationship) can be achieved by a different, larger feedforward computation (Doerig et al., 2019). Thus, the authors argue that causal structure theories such as ITT (and the TICL) are either falsified or non-scientific. Falsification would occur if causal structure theorists allowed that feedforward structures of causality could be conscious (Doerig et al., 2019). Given that Doerig et al. limit the scientific evidence for consciousness to the subjective report of content, it appears to be impossible to determine whether any other person or thing is conscious. Rene Descartes grounded his philosophy in the undeniable fact of his own consciousness. Descartes wrote, “…this truth, I think hence I am, was so certain and of such evidence, that no ground of doubt, however extravagant, could be alleged by the skeptics capable of shaking it, I concluded that I might, without scruple accept it as the first principle of the philosophy of which I was in search” (Descartes, 1912). Citing Descartes, Tononi and colleagues suggest, axiomatically, that conscious experience exists intrinsically, which is to say it exists to itself (Tononi et al., 2016). In my opinion, this is a misunderstanding that leads to errors in IIT. In describing himself as a “thinking thing”, Descartes is not necessitating that he, the thing with thoughts, and the thoughts which he is thinking are one and the same thing. It seems apparent to me, following Descartes, that the thoughts are intrinsic to the thinker, or “thinking thing” that is conscious. Descartes infers his own existence from that of his thoughts. In fact, he does not exist to himself. Rather, his thoughts exist to him. In recognition of this, the TICL posits that the Systemic TIC is aware of the existence of its Subsystemic TICs. The latter are intrinsic to the former. Thus, we see and feel and think about contents, but we can only infer our own existence from those contents. It is rare for neuroscientific theories of consciousness to explicitly address the point of view. However, ultimately, the point of view is what we are seeking to explain.

#### Consciousness Is Temporally Continuous

Fourth, consciousness is temporally continuous (Wittmann, 2011; Winters, 2020; Kent and Wittmann, 2021). John Searle defined consciousness as “those states of sentience and awareness that typically begin when we awake from a dreamless sleep and continue until we go to sleep again, or fall into a coma or die or otherwise become unconscious” (Searle, 1997). Phenomenologically, we experience no borders between subsequent experiences. In “The Principles of Psychology” (1890) William James said, “Consciousness does not appear to itself chopped up in bits. Such words as “chain” or “train” do not describe it fitly as it presents itself in the first instance. It is nothing jointed; it flows. A “river” or a “stream” are the metaphors by which it is most naturally described. In talking of it hereafter, let us call it the stream of thought, of consciousness, or of subjective life”. James recognized that, within a conscious experience, we observe change occurring in a non-discrete, but continuous manner. This may reflect the nestedness of qualitative contents occurring at different temporal scales (Fingelkurts et al., 2010). Whether this necessitates a non-discrete mechanistic correlate in the brain remains a matter of contention (Fingelkurts et al., 2010; Kent et al., 2019; Winters, 2020; Kent and Wittmann, 2021). Nevertheless, contents are dynamic and consciousness seems to flow over an extended sense of the present (Poppel, 1997; Kent et al., 2019).

#### Consciousness Is Limited and Coherent

Finally, consciousness is limited and coherent. At any given time, most things that could be conscious are not. While the conscious composition contains many simultaneous contents, incoming sensory data streams are mostly unnoticed. Thus, only limited content is subjectively accessible (Dehaene and Changeux, 2011). The contents of consciousness are limited to a subset, and this suggests a threshold for perception. Furthermore, only a single interpretation of contents exists from our point of view at any one time (Blake and Logothetis, 2002; Tsuchiya and Koch, 2005; Imamoglu et al., 2012). This is well demonstrated by visual illusions such as the Necker cube, and by binocular rivalry. The neural correlates of limited conscious content are studied using contrastive analysis at liminal thresholds for perception.

These five phenomenal aspects are derived from human consciousness. We have no way of knowing whether they are fundamental to consciousness itself. The TICL attempts to explain human phenomenal consciousness as a landscape of nested EM field structures. The neural correlates of human consciousness have evolved over millennia, and do not provide insight into the simplest, most primitive, modes of conscious being. Further, there is debate among theorists as to what the key phenomenal features of human consciousness are. The five axioms of IIT overlap with those presented here, but they are distinguishable at least in the case of intrinsicality and exclusion (Tononi et al., 2016; Winters, 2020). With respect to intrinsicality, an area of contention among current frameworks, often implicitly, is the view that consciousness is one thing altogether and intrinsic to itself (Tononi et al., 2016) rather than one thing containing many nested things intrinsic to and differentiated within it (Fingelkurts et al., 2010, 2013; Northoff and Huang, 2017; Winters, 2020). It is my view, that the latter is a closer approximation to the phenomenal human experience. It is often unclear where a theoretical framework falls on this question. The TICL explicitly accounts for the point of view as being that of the wider, integrated System upon its internal dynamics (Winters, 2020). With respect to exclusion, IIT posits that conscious experience has one, definite spatial and temporal grain (Tononi et al., 2016), which contrasts with the view that human consciousness is temporally continuous with dynamic contents nested within it (Fingelkurts et al., 2010, 2013; Wittmann, 2011; Northoff and Huang, 2017; Winters, 2020; Kent and Wittmann, 2021).

My goal in developing the TICL was to establish a framework in which the phenomenal aspects of human consciousness are in parsimonious agreement with their neural (and ultimately physical) correlates. I suggest that the five fundamental features of human consciousness named above require a general physical model of consciousness to take the form of a single, integrated thalamocortical structure corresponding to the state of consciousness, within which differentiated neural activities are nested in space and time, with the limitation and coherence of conscious contents depending on the perceived distinction between background and the differentiated ensembles from the point of view of the unified structure, and with the meaning being intrinsic to the relationship among neural activities from that point of view (Winters, 2020).

The TICL posits a general explanation for the five fundamental aspects of phenomenal consciousness I have presented. Human consciousness is a unified composition of contents because the Subsystems occur within the unified (physically integrated) System. The contents of consciousness are specific because the Subsystems are composed of specific neuronal elements and their specific TIC. They are meaningful because of their relationship to one another and to the System. The contents exist from the point of view of the System. The System remains largely unchanged in spatial and temporal terms, but it persists in time (temporal continuity) as Subsystems appear, change, and disappear within it, in their own time. Finally, the TICL is limited by the necessity of Subsystems to have a higher level of TIC than the System, making them distinguishable from background noise, and thus meaningful to the System. Coherence is achieved because Subsystems cannot have more than one form (meaning) at the same time, from the Systemic point of view. Like other leading neuroscientific theories, the TICL is consistent with a large body of experimental evidence.

### The Neural Correlates of Consciousness in Brief Review

The mammalian brain sustains states of consciousness during wakefulness and dreaming sleep, but these are abbreviated by states of nonconsciousness during non-dreaming sleep. This state-change requires enabling factors centered in the brainstem and acting widely across the rest of the brain (Parvizi and Damasio, 2001). During conscious states, whether waking or dreaming, cortical EEG shows asynchronous, high-frequency activity (Siclari et al., 2017). Spontaneous oscillations in the cortical EEG occur when a large number of neurons are acting in concert (Steriade et al., 1990). These EEG rhythms are classically distinguished as delta (1–3 Hz), theta (4–8 Hz), alpha (9–12 Hz), beta (13–30 Hz), and gamma (>30 Hz). It has been suggested that synchronization of neuronal activity, in the gamma frequency band, enables temporal coordination between the large number of inputs and the resulting outputs (Fries, 2015). Such synchronized oscillations co-exist in the brain with arrhythmic scale-free activities in which subsets of neurons fire in synchrony but not in a periodic fashion (Freeman, 2005; Thivierge and Cisek, 2008; Milstein et al., 2009). The scale-free dynamics of human brain activity, in EEG, are characterized by considerable nesting of frequencies (He et al., 2010). The phase of lower frequencies modulates the amplitude of higher frequency neuronal activities (He et al., 2010) in a manner known as cross-frequency coupling, in which small, local populations of neurons are influenced by the low-frequency oscillations occurring over larger populations (Bragin et al., 1995; Canolty et al., 2006; Canolty and Knight, 2010; Aru et al., 2015). Cross-frequency coupling has been suggested to be involved in information exchange and cognitive processes (Tort et al., 2009; Axmacher et al., 2010; Canolty and Knight, 2010; Lisman and Jensen, 2013).

The contents of consciousness are generally understood to be generated by activity limited to a large portion of the cerebral cortex and the thalamus (Koch et al., 2016; Tononi et al., 2016). Primary cortical structures, such as V1, do not directly contribute to consciousness (Weiskrantz, 1996; Lamme and Roelfsema, 2000; He and MacLeod, 2001; Jiang et al., 2007). The cortex is very complex, but it is orderly, with hierarchical processing of incoming data streams from primary modules to higher, association modules. Network modules are subsets of neurons or neuronal groups that are highly connected to one another (Bassett and Sporns, 2017). These overlapping and non-overlapping subsets of nodes in the network are strongly connected to one another but only weakly connected to the wider network (Sporns and Betzel, 2016). Highly connected brain networks along the midline have been described as connector hubs with widespread regional connections by means of cortico-cortical axonal pathways (Hagmann et al., 2008). Modulation across different anatomical networks is arranged hierarchically (Sadaghiani et al., 2010). Dynamic changes in synchrony might drive the capacity for groups of neurons to coalesce into functionally connected ensembles (Fries, 2015). Interestingly, a hierarchy of timescales has been described, noting that association areas, further along a sensory pathway become selectively activated with stimuli that are coherent over longer time periods (Hasson et al., 2008; Murray et al., 2014). In the spatial domain, such hierarchies are apparent in the visual system, in which receptive field sizes increase along the visual pathway. A hierarchy of timescales may be involved in functional specialization across the cortex (Buzsáki and Draguhn, 2004; Murray et al., 2014). Often no direct structural connection is apparent between populations, though they function coherently with one another (Honey et al., 2009). Accordingly, long-range relationships among spatially distributed regions, have been discovered (Sporns and Betzel, 2016). Examples include the frontoparietal control network and the default mode network (Power et al., 2011). States of nonconsciousness are characterized by reduced functional connectivity across the cerebral cortex and a loss in the diversity of connected configurations (Mashour and Hudetz, 2018).

Christof Koch distinguishes the full neural correlates of consciousness (NCC) and the content-specific neural correlates of consciousness (content-specific NCC; Crick and Koch, 1998; Koch et al., 2016). The former (NCC) are the total necessary and sufficient activities in the brain for the production of consciousness, without regard to particular contents. The content-specific NCC refers to the total necessary and sufficient neural activities for the production of consciousness with a given piece of content (Koch et al., 2016). Distributed neural activities across the cerebral cortex are unified by means of functional integration (Massimini et al., 2005; Boly et al., 2012; Hudetz, 2012; King et al., 2013; Monti et al., 2013; Marinazzo et al., 2014; Tagliazucchi and Laufs, 2014; Tononi et al., 2016; Mashour and Hudetz, 2018). This has been proposed to depend on re-entry, recurrent loops or feedback communication between cortical regions (Tononi and Edelman, 1998; Lamme and Roelfsema, 2000; Dehaene and Naccache, 2001; Supèr et al., 2001; Dehaene and Changeux, 2011; Oizumi et al., 2014). An alternative mechanism for integration is EM resonance (Hunt and Schooler, 2019).

Ongoing neural activity across the thalamocortical system occurs at a range of spatial and temporal scales (Sadaghiani et al., 2010). Distinct frequency bands are modulated over time with a predominance of slow-wave activity (Leopold et al., 2003; Nir et al., 2008). Higher frequency activity is nested into the infra-slow fluctuations, which occur at less than 0.1 Hz (He et al., 2010). These especially slow oscillations occur over long cortical distances with wide spatial coherence even across cerebral hemispheres (Leopold et al., 2003; Nir et al., 2008). Ongoing, slow fluctuations have also been observed in fMRI, with coherence across wide ranges providing evidence for functional connectivity (Shmuel and Leopold, 2008).

The content-specific NCC can be studied in laboratory settings using controlled, sensory stimuli. It has largely been accomplished utilizing report-based visual paradigms and has identified the involvement of both frontal and parietal regions (Breitmeyer and Ogmen, 2000; Blake and Logothetis, 2002; Tsuchiya and Koch, 2005; Imamoglu et al., 2012). Similar studies which avoid overt reports of perception suggest that the content-specific NCC are limited to only posterior cortical regions (Frässle et al., 2014; Tsuchiya et al., 2015). The matter is far from settled and probably depends upon experimental approaches and phenomenal definitions as much as it does upon contradictory evidence. The results of transcranial magnetic stimulation studies with EEG in conscious and non-conscious subjects are strongly suggestive of differential oscillations across space being a specific feature of the conscious state (Massimini et al., 2005; Sarasso et al., 2015). The idea that both large-scale integration and smaller-scale differentiation are necessary for the consciousness of content may have first been recognized in the Dynamic Core Hypothesis (Tononi and Edelman, 1998). Neuronal oscillations temporally link neurons into ensembles by means of synchrony (Buzsáki and Draguhn, 2004). Local synchrony at high frequencies may bind features of conscious percepts together but it also occurs among groups of neurons in cases where stimuli are not consciously perceived (Ray and Maunsell, 2010; Pitts et al., 2014; Hermes et al., 2015). Temporo-spatial nestedness has been proposed to correlate with the neural predisposition to the consciousness of stimuli (Northoff and Huang, 2017).

Experiments have shown that increased phase synchrony over long distances, in the cortex, correlates with conscious perception of stimuli (Gross et al., 2004; Gaillard et al., 2009). Localized increases in gamma power and synchrony are seen even with stimuli that are not consciously perceived, particularly within the first 200 ms (Melloni et al., 2007; Ray and Maunsell, 2010). Despite this, most theoretical frameworks for consciousness limit the temporal aspects of consciousness to a few hundred ms timescales (Northoff and Lamme, 2020; Kent and Wittmann, 2021). Phenomenologically, it has been suggested that the experienced present moment is actually occurring over a wider temporal window in which the contents of consciousness are integrated (Poppel, 1997; Kent et al., 2019; Kent and Wittmann, 2021). A large number of studies have shown that the brain’s spontaneous activity during conscious states, prior to an experimental stimulus, is relevant to the resulting conscious content (Northoff and Huang, 2017). With weak stimuli (just at threshold), the presentation of which will sometimes result in perception and sometimes not, within the same subject, baseline, or resting state, activity as measured by fMRI, positively correlates with conscious perception (Boly et al., 2007; Hesselmann et al., 2008; Ploner et al., 2010; Qin et al., 2016). Furthermore, the phase of the cortical alpha rhythm, measured by EEG is also predictive of whether a stimulus will be perceived, with significantly lower detection of the stimulus during the trough of the alpha band than during the peak (Mathewson et al., 2009). It was shown, using magnetoencephalography (MEG), that pre-stimulus alpha fluctuations predict the capacity to visually discriminate (Van Dijk et al., 2008). These findings are consistent with the idea that temporal alignment between background oscillations and stimulus-driven activities determines whether stimuli are consciously perceived (Northoff and Huang, 2017). A further temporal feature of conscious perception is informed by visual studies on “masking”. Brief visual stimuli which, presented by themselves, are perceived by the subject, can be rendered unperceived by spatially and temporally adjacent stimuli (“masks”) even when they are presented after the initial stimulus (Breitmeyer and Ogmen, 2000; Dehaene and Changeux, 2011). This suggests that conscious contents are not evaluated in immediate sequence, but according to a wider temporal window (Kent and Wittmann, 2021).

Rather than disputing the credibility of these experimental results, theories of consciousness differ in their interpretation of them and preferentially address certain areas of evidence. I described five aspects of phenomenology that characterize human consciousness. I said that human phenomenal consciousness is: (1) unified and compositional; its contents are (2) specific and meaningful; and (3) they exist from a subjective point of view. Consciousness is (4) temporally continuous; and (5) limited and coherent. Thus, differing theoretical frameworks can be distinguished by their particular explanations for these phenomenal features. A variety of theoretical frameworks have proliferated recently, and different aspects of consciousness explored using different experimental methods might account, in large part, for the discrepancies among them (Northoff and Lamme, 2020). Neuroscientific theories of consciousness contrast along multiple dimensions. For better or worse, the recent proliferation of theories has often meant differing vocabularies to describe overlapping or identical concepts. It is, thus, worth attempting to distill the fundamental ideas presented by the theorists in order to undertake their comparison. This leads to an imperfect but useful mapping of the relations among concepts that make up the theories. Entrenched theorists risk talking past one another. It is my hope that following the expansion of theoretical models, an evidence-based convergence will ultimately take hold as the field matures. Therefore, I will take a reconciliatory approach as well as a discriminating one, in this discussion.

### The Fundamentals of Neuroscientific Theories of Consciousness

#### Integrated Information Theory

Integrated Information Theory (IIT) begins with a set of axioms, or self-evident phenomenal facts about consciousness, and derives postulates about the physical substrate of consciousness. In brief, these are the axiom of intrinsicality, the axiom of composition, the axiom of information, the axiom of integration, and the axiom of exclusion (Tononi et al., 2016). There is considerable overlap between these axioms and the five aspects of consciousness that I have highlighted in the present article. Reasoning from these axioms, IIT predicts that the physical substrate of consciousness must be the maximum of intrinsic cause-effect power in the thalamocortical system (Tononi et al., 2016). According to IIT, conscious entities are not temporally or spatially nested structures. Rather consciousness is intrinsic to the system of elements across which the maximum of cause-effect power is occurring, given a time constant, at or around 200 ms (Tononi et al., 2016). Thus, for IIT, the unified conscious mind is a single, discrete structure of integrated information with the content of the whole specified by the structure (Tononi et al., 2016). Dynamic, nested Subsystems are excluded from consciousness, though more than one conscious entity might share the brain at a given time in a non-spatially-overlapping arrangement (Oizumi et al., 2014). I predict that the maximal cause-effect power over a set of neuronal elements at a given timescale in the thalamocortical system might correspond to its most salient content at the moment of measurement, rather than capturing consciousness with all of its ongoing content.

#### Global Neuronal Workspace Theory

Global Neuronal Workspace (GNW) posits that “conscious access” is a means by which information is widely spread through the cerebral cortex (Dehaene and Changeux, 2011). This is suggested to occur by means of delayed amplification of sensory network activity, which leads to long-range synchronization in beta and gamma frequencies (Dehaene and Changeux, 2011). Once access has been achieved and communication is occurring across the “global workspace”, information becomes unified into a common conscious mind (Seth et al., 2005). According to GNW, cortical pyramidal neurons and their related thalamocortical loops are functionally interconnected to form a “global workspace”. Reciprocal connections among local, specialized modules enable contents to be united into a common structure (Baars, 2005; Dehaene and Changeux, 2011). GNW theorists call this communication a broadcast because it spreads information from, for example, parietal and temporal modules to prefrontal cortical ones, and subsequently makes cognitive recognition and verbal report possible (Dehaene and Naccache, 2001; Dehaene and Changeux, 2011). “Conscious access” provides a threshold mechanism for unconscious neural events to be made conscious. It would appear that this may be consistent with nestedness in space and time, even if the idea has not been claimed explicitly.

#### General Resonance Theory

According to General Resonance Theory (GRT), shared resonance, an idea related to functional coherence, combines micro-conscious entities into macro-conscious ones (Hunt and Schooler, 2019). Despite the panpsychist framing of GRT, the existence of micro-conscious entities is not dissimilar from the claim, made by IIT theorists, that structures which exhibit some measure of integrated information, no matter how small its manifestation, may have some level of consciousness (Tononi and Koch, 2015). Similarly, the concept of “conscious access”, in GNW, while not explicitly an answer to the combination problem, points to a parallel problem of unified consciousness (Dehaene et al., 2014). How do distal networks communicate across the brain, toward the production of conscious contents? The answer, for GRT, lies in the synchronization of their activities into a common system. Its proponents suggest that the brain’s EM fields make this resonance possible (Hunt and Schooler, 2019). They point to a hierarchy of resonances in the brain, in accordance with varying oscillations in the brain occurring on a background of non-oscillating, low-frequency activity (Steinke and Galan, 2011). Resonance, or synchrony, among neural populations is proposed to be driven by electrical fields. Despite this mechanistic novelty, the GRT says that “dominant consciousness”, the conscious entity exhibited as the human mind, is unified by synchrony into a single system (Hunt and Schooler, 2019). It follows that the contents of “dominant consciousness” are intrinsic to it, much as the maximum of the cause-effect power structure of IIT. Hunt and Schooler propose that a phase transition occurs to facilitate the efficient, high-speed flow of information, reminiscent of the “ignition” discussed in GNW. The content of consciousness is spatially determined by the set of neurons which are in resonance. Even though the theory allows for nested micro-conscious entities occurring at multiple causal speeds, it is unclear if GRT allows for multiple different resonances to co-exist in the “dominant consciousness”. I have been arguing that the contents of consciousness are nested within the conscious System, with independent Subsystemic dynamics and independent Subsystemic synchronies. If GRT lacks multiple-resonance-frequency dynamics, this idea appears to contrast with GRT as completely as it does with IIT.

#### Operational Architectonics

Operational Architectonics (A-O) purports that unified consciousness is achieved by means of a dynamic, nested hierarchy of electromagnetic fields in the brain (Fingelkurts et al., 2010, 2019). An internal physical space-time (IPST) reorganizes and processes signals from the outside world, external physical space-time (EPST), turning those streams of data into dynamic, volumetric spatial-temporal patterns of local extracellular electric fields (Fingelkurts et al., 2010). These EM fields, or operational modules, exhibit intrinsic phenomenal character. This amounts to a virtual world for the subject known as phenomenal space-time (PST; Fingelkurts et al., 2019). Short–term patterns of integrated activity occur within the IPST and become unified within the PST (Fingelkurts et al., 2010). This framework is undeniably one of nestedness. Assemblies of neurons take in energy over time, which is suggested to suddenly offload entropy by means of a rapid transitional process, which then reorganizes the whole system and allows the intermittent emergence of new content in PST (Fingelkurts et al., 2013). There is a recognition of dynamic content occurring within a wider frame of nested assemblies (thus nested EM fields), which suggests a high degree of reconcilability with the TICL.

#### Temporo-spatial Theory of Consciousness

The Temporo-spatial Theory of Consciousness (TTC) frames the problem of consciousness in terms of four dimensions, or aspects, and offers a set of solutions (Northoff and Huang, 2017). These are: (1) the level or state of consciousness; (2) the content or form of consciousness; (3) phenomenology or experience; and (4) cognitive processing and report. First, the level or state of consciousness is a predisposition to the consciousness of content which corresponds with temporo-spatial nestedness or neural activity. This, of course, is directly relevant to the current discussion and relies upon a large body of evidence reviewed, in part, above. According to the TTC, nestedness in space and time is critical to the state of being conscious. It represents a readiness for stimuli to become consciously perceived (Northoff and Huang, 2017). The authors relate temporo-spatial nestedness to the “dynamic repertoire”, which refers to the temporal and spatial range of neural reactions that occurs in conscious states but is substantially reduced in non-conscious states (Hudetz et al., 2015). The contents of consciousness (dimension 2) are related to temporo-spatial nestedness in terms of alignment between stimulus time and strength with the underlying oscillations. It is referred to as the neural prerequisite of consciousness (Northoff and Huang, 2017). Slow-wave activities are hypothesized to provide a temporal window in which the network is receptive to the integration of stimulus-induced activity (Hasson et al., 2008). With respect to phenomenology or experience, TTC connects this to the spatial and temporal expansion of stimulus-induced activities (Northoff and Huang, 2017). The stimulus-driven activity becomes integrated across the brain but is differentiated in terms of its spatial and temporal configuration (Northoff and Huang, 2017). Dimension 4 of the TTC deals with cognitive processing and report and thus reflects delayed stimulus-driven effects (Northoff and Huang, 2017).

### Competing Theories Offer Different Explanations for Phenomenal Features of Human Consciousness

#### What Unifies Consciousness?

**Figure 2:**
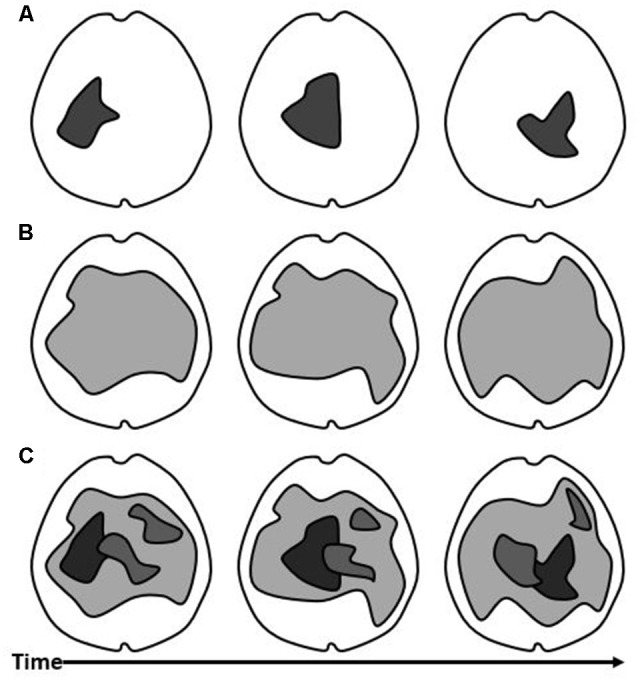
*Models of Consciousness. The thalamocortical substrate of consciousness, proposed by different theories can be generally mapped onto these models*. TIC is represented as the darkness of the color gray. **(A)** In this model, the substrate of consciousness is a single, highly integrated network in the thalamocortical system. Over time, its spatial character and position in the brain changes (different shapes at different positions in the three panels). **(B)** In this model, the substrate of consciousness is a single, large, and integrated or resonant network in the thalamocortical system. Its elemental make-up changes over time (gray shape over three panels). **(C)** In this third model, the substrate of consciousness consists of a large, integrated network in the thalamocortical system (light gray), which can undergo some elemental change across time (shape across panels). Nested within this network are smaller networks with higher TIC (darker colors).

Since human consciousness is composed of contents of various types which manifest as a unity, it is necessary for a theory of consciousness to explain how this is accomplished in the brain. Figure 2 shows three different models which broadly represent the different theoretical ways of handling unity. According to IIT, unity occurs by means of network integration in the maximal cause-effect structure over a precise spatial and temporal frame (Tononi et al., 2016). This is a kind of causal integration, assumed to occur among neuronal elements by means of mutual influence. In that sense, it is very similar to the assumptions of the TICL, which unifies the contents of consciousness within a common structure of causality, in a nested way. The unity for IIT, however, is proposed to be quite limited in spatial and temporal breadth, as in Figure 2A. Like in Figure 1, the degree of integrated causality, information exchange, or functional synchrony is represented by the darkness of the gray color. In Figure 1A, we see a single, highly integrated structure (dark gray), as proposed to be the physical substrate of consciousness in IIT. According to GNW, The global workspace unifies the content by making information widely available (Dehaene and Naccache, 2001). This idea is mechanistically distinct but still seems to allow that the communication of information across a common, integrated structure is key to unifying the content. The spatial location in the brain where this takes place is understood in GNW to include prefrontal cortical structures, and the timing with respect to stimulus-onset to conscious perception is late relative to IIT. In a previous article, I criticized GNW as potentially situating the conscious “global workspace” separately in space from the cortical networks responsible for the content (Winters, 2020). This interpretation would have GNW being represented by Figure 2A, like IIT. The difference would be that, for GNW, the substrate of consciousness would be situated further toward the front of the cortex. While that interpretation of the “global workspace” might be incompatible with the TICL, reconciliation could be had by understanding that the “global workspace” consists of the posterior cortical networks producing content as well as the frontal cortical workspace neurons, as long as they are in sufficient communication. In the latter case, GNW might look more like Figure 2C, wherein content-producing networks would be nested within the “global workspace”. For GRT, spatially widespread synchronization by means of EM fields, unifies consciousness. Those neuronal constituents which are part of the “dominant consciousness” make up its unified content (Hunt and Schooler, 2019). This might be best illustrated by Figure 2B, wherein the spatial domain of the “dominant consciousness” is larger than IIT’s maximum of cause-effect power. In O-A, phenomenal space-time is a subjective “virtual world” in which nested, local EM field activity self-presents (Fingelkurts et al., 2010). This is best represented by Figure 2C. The latter is more consistent with the TICL, in that the neuronal ensembles responsible for content are nested in space and time within a common EM field. The difference is one of explicit terminology regarding consciousness in the brain as an integrated structure of electromagnetism vs. an integrated structure of causality. Reconciliation between theories is possible, once we recognize that EM forces are necessarily the mechanism of causality instantiated in the brain, in the TICL, or any other neuroscientific theory. According to TTC, spatial-temporal nestedness of integrated activity occurring upon the brain’s spontaneous activity, as in cross-frequency coupling, unites the contents of consciousness (Northoff and Huang, 2017). Again, this model is consistent with Figure 2C. This proposal satisfies the claims of the TICL framework but explores the spatial and temporal mechanisms of integration much more specifically. Figure 2C is an appropriate model for how the TICL accounts for the unity of conscious contents. In Figures 2A,B, the structure of consciousness is one thing altogether. By contrast, Figure 2C allows for the spatial and temporal nesting of content-producing neural structures within a hierarchical arrangement. Notably, the dark gray shape appearing in Figure 2A is contained within the larger, light gray shape in Figure 2C. This reflects that, in the TICL, the maximum cause-effect structure is a Subsystem contained within a larger System (Winters, 2020).

#### What Specifies the Content?

The contents of consciousness are specific and qualitatively distinguishable. They tend to arise in correlation with localized cortical functions in a hierarchical arrangement. According to IIT, the specificity of content is given by the constituent elements of the substrate of consciousness and their causal power. The contents are built into the maximal cause-effect structure (Tononi et al., 2016). Thus, Figure 2A shows a single structure in the brain corresponding to a high level of integrated causality. The precise structure of cause and effect which currently composes the maximum, is unique and different from any other configuration, allowing for unimaginable variety (Tononi et al., 2016). According to the GNW, specific contents occur due to the sustained activity of a fraction of thalamocortical neurons which are broadcast to, or “accessed” by the “global workspace” (Dehaene and Naccache, 2001; Dehaene and Changeux, 2011). Put side-by-side, this is not so different from IIT, in principle. Which neurons are integrated or in communication, determines the qualitative character of consciousness, in both models. The TICL is roughly compatible with this. In the TICL, a Subsystem is a subset of neuronal elements within the spatially and temporally wider integrated System, which is even more causally-integrated over a shorter timeframe than the whole System (Winters, 2020). The specificity of content is given by the composition of Subsystems which exist at a given moment, and how they are changing. In Figure 2C, the darker shapes within the lighter gray shape, correspond, in simplified form, to a set of Subsystems. For GRT, the contents are specified by the particular constituent neurons in the resonant structure (Hunt and Schooler, 2019). Thus, in Figure 2B, there are no separate shapes distinguished within the single, common neural substrate of consciousness. In O-A, local extracellular EM fields are highly structured in space-time. This structure determines the content of PST (Fingelkurts et al., 2010). The combinatorial power within and among operational modules enables near infinitely diverse qualitative contents (Benedetti et al., 2010). The operational modules are similar to the concept of Subsystems, and they appear something like the image in Figure 2C. According to TTC, the stimulus-induced high-frequency activity becomes expanded, similar to “conscious access” in GNW. This integrates the nested activity within the context of the brain’s spontaneous activity (Massimini et al., 2005; Northoff and Huang, 2017). The specificity comes from its spatial-temporal configuration within the integrated brain (Northoff and Huang, 2017). This is well illustrated in Figure 2C. Each in its own terms, these frameworks come to fairly similar conclusions about specificity. In the thalamocortical system, a large variety of functional configurations are possible at any given time. One way or another, this enables a wide range of contents.

#### What Is the Point of View?

According to IIT, consciousness is intrinsic to itself. It is identical to the collected content and the point of view upon it (Tononi et al., 2016). A conceptual difficulty for this viewpoint is that consciousness cannot be continuous if its substrate is not the same thing from instance to instance (Figure 2A across time). In fact, it seems to me that a conscious being would be a brief moment of existence, with an endless stream of new conscious beings existing one after another. This is at odds with phenomenology. In GRT, “Dominant consciousness” must be intrinsic to itself, as well. If Figure 2B is a fair representation of the spatial domain of the resonant structure, it must provide its own intrinsic point of view. Like in IIT, the structure of consciousness must be understood to exist to itself. By contrast, for GNW, the “global workspace” must provide the point of view upon the content to which it has access. This is more like the TICL, in which the System has a point of view upon the character and dynamics of its subsystems (Winters, 2020). The point of view is understood to be the thing which contains the content. This insight reverses the normal perspective of feeling as if we look out upon the world since the phenomenal world occurs *within* consciousness. The caveat for GNW is the one which I mentioned above; the “global workspace” which achieves the nesting of contents within consciousness must include the cortical regions which produce the content. If it does this, then the “global workspace” can provide the point of view upon the contents which occur with the sustained, but limited, thalamocortical activity which is being accessed in consciousness. Thus, GNW might look something like Figure 2A except that the structure of consciousness would have a more stable anatomical shape and would be situated more frontally. Alternatively, as suggested above, GNW might be interpreted closer to what is seen in Figure 2C, in which the point of view is that larger, lighter gray structure containing the smaller, darker ones. In O-A, PST is a highly abstract self-presentation of operational spatial-temporal patterns (Fingelkurts et al., 2010). It follows that the PST is the point of view upon those operational patterns. As long as that is the understanding, then the framing provided in the TICL is perfectly amenable to O-A, with the larger, lighter shape providing the point of view upon its contents (Figure 2C). While not stated explicitly in the TTC literature, it might be that the point of view is the widest spatial-temporal integrated structure, as in the TICL. For TTC, the brain’s intrinsic space and time are given by the spatial extension and temporal duration of neural activities (Northoff and Huang, 2017). If the authors’ viewpoint is consistent with the TICL, then the wider extension in space and time corresponds to the System of TICL and the theories are compatible (Winters, 2020). For the TICL, the System (lighter gray) contains specific Subsystems (darker gray shapes), the activities of which it experiences from its own, larger point of view.

#### How Is Conscious Continuity Understood?

According to IIT, consciousness is a sequence of discrete instances, each replacing the former, not continuous (Tononi et al., 2016). This has been criticized as a difficulty for IIT because the theory begins with a set of self-evident axioms without acknowledging temporal continuity (Wittmann, 2011; Winters, 2020; Kent and Wittmann, 2021). We see that in Figure 2A, across the three points in time, the physical substrate of consciousness has changed substantially. For IIT, this is not a gradual morphing, but a sequence of structures gaining ascendency. By contrast, GNW supports this feature of phenomenal consciousness. According to GNW, the sustained activity of thalamocortical neurons should allow a period of continuity for cognitive utilization, even for seconds after the disappearance of the immediate stimulus activity (Dehaene and Naccache, 2001; Baars, 2005). The information which is globally available should be updated as a continuous stream (Dehaene and Changeux, 2005). The integration of experience, for GNW, occurs both at a point in time and across time (Mashour et al., 2020). In GRT, the borders of the “dominant consciousness” are continually changing as activities come into resonance with them, by undergoing phase transition (Hunt and Schooler, 2019). However, the dynamics of conscious content must be encoded spatially because there is no difference in temporal character; they are synchronous (McFadden, 2020). This is what we see in Figure 2B over the three time points. In O-A, dynamic content exhibits intermittence because of the suggested rapid transitional process and subsequent restructuring (Fingelkurts et al., 2013). But, phenomenal space time consists of spatially and temporally nested content (Fingelkurts et al., 2010). This implies that O-A is capable of supporting both temporal continuity of the conscious state as well as dynamic contents within conscious experience. This is illustrated in Figure 2C, in which the networks responsible for content are changing across time. According to TTC, temporal receptive windows are arranged hierarchically (Northoff and Huang, 2017). This theory also exhibits temporal continuity with overlapping and nested contents in time. In Figure 2C across the time points, we see changes in the nested content-producing networks as well as spatial overlap. Thus, IIT stands alone among these theories in insisting upon a discrete timeframe for the entire conscious experience. The TICL, as well as O-A and TTC in particular, proposes continuous consciousness with nested contents in space and time. For the TICL, Figure 2C shows that the System is continuous across time, even as it may undergo some spatial change as elements enter and exit the integrated structure. Meanwhile, Subsystems can arise, change, and disappear. Notice that the Subsystems are always shown in darker gray to reflect that they must have a higher level of TIC than the System in which they are embedded. Also, notice that the most highly integrated Subsystem (darkest gray object) is that which appears in Figure 2A. The structure which IIT purports to be the whole substrate of consciousness should correspond to a high-TIC Subsystem for TICL, contained within the lower-TIC, but spatially and temporally larger, System.

#### How Is Consciousness Limited?

In IIT, contents are only possible where their underlying elemental activities are within the spatio-temporal borders of the maximally irreducible cause-effect structure (Tononi et al., 2016). This means that most neurons in the integrated thalamocortical system are not producing content at a given time. Thus, in Figure 2A, most of the brain is shown in white. Similarly, in GRT, the borders of the “dominant consciousness” are limited in accordance with the neurons which are resonant or synchronized (Hunt and Schooler, 2019). This is roughly what is shown in Figure 2B, with much of the thalamocortical system in “dominant consciousness”. In GNW, long-range synchronization facilitates “conscious access” by the “global workspace” (Dehaene and Changeux, 2011). Only a limited fraction of thalamocortical neurons are “ignited” and sustained, providing internal coherence, while the rest are inhibited (Dehaene et al., 2003; Dehaene and Changeux, 2011). According to O-A, local extracellular EM fields are perceivable as nested within the wider EM field (Fingelkurts et al., 2019). Presumably, a threshold is determined by the strength of local extracellular EM fields, such that too weak a field is unperceivable. TTC proposes a mechanism of alignment between stimulus time with phase preference to the underlying spontaneous activity and a threshold driven by resulting neural amplitude (Northoff and Huang, 2017). For the TICL, the presence or absence of a Subsystem depends upon the subset of neuronal elements which would make it up having a higher degree of TIC than the larger System does (Winters, 2020). The content is limited to the Subsystems which exist at a given time. Any subset of neurons which is exhibiting causality in the integrated System, but not to a greater degree than the System, is buried in the noise, unmeaningful, and not experienced (Figure 2C in light gray).

## Conclusions

The TICL makes claims that distinguish it in the field of theoretical frameworks. The TICL builds its foundation upon five phenomenal aspects of human consciousness, with the assumption that the most parsimonious explanation for these phenomenal aspects will be an arrangement of physical structure and interactions (anatomy and physiology) which mirror them. Descartes wrote, “…this truth, I think hence I am, was so certain and of such evidence, that no ground of doubt, however extravagant, could be alleged by the skeptics capable of shaking it, I concluded that I might, without scruple accept it as the first principle of the philosophy of which I was in search.” (Descartes, 1912). Thus, he observed content and inferred that he must exist. He asked himself what he is and concluded that he is a “thinking thing”, a thing with thoughts (and perceptions). Whatever conscious being is, it is a point of view upon contents. With this undeniable fact in mind, we can make observations about the contents of consciousness, from which we note that they are specific, limited, and meaningful, and that they are continually changing. They are specific and meaningful in that we can distinguish among them (sound vs. image, left vs. right, blue vs. red). They are limited in that we do not experience all of the potential contents all the time. And, they are dynamic. The point of view persists as the contents change. This results in a model in which the unified mind (and therefore integrated brain function; a System) contains phenomenal contents within it (differentiated Subsystems). Descartes’ dualism made the assumption that the contents were real things (physical stuff) and that consciousness was a separate real thing (mental stuff). A physical, scientific account of phenomenal consciousness must reject this separation. By nesting the content-specific NCC within the full NCC, we arrive at a common structure of which the point of view must be that of the full NCC upon the content-specific NCC (Figure 1). The neural correlate of the point of view which contains the content-specific NCC must be not only spatially larger but also temporally longer. By this means, the point of view can bear witness to changing content. According to the TICL, consciousness is a complex structure of integrated causality in time. Causality necessitates force, and EM (Lorentz force) is almost certainly the force at play. The distinctions I wish to highlight among the theories discussed here are not drawn between those which are explicitly network-based (IIT, GNW, TCC) and those which are explicitly EM field-based (GRT and O-A). Rather, the critical distinction is between those theories in which the content-specific NCC are nested within the full NCC and those which conflate the two. In my general analysis, the TICL, O-A, and the TTC best exemplify this distinction. Critically, these explicit mechanisms for the neural correlates of consciousness ultimately collapse into a common implicit mechanism: some arrangement of EM interactions. Recognizing this will enable theoretical neuroscience to escape the bounds of biological and psychological thinking and place our deepest problem (the problem of consciousness) firmly within the purview of physics, where an explanation, after all, will be elucidated.

## Data Availability Statement

The original contributions presented in the study are included in the article, further inquiries can be directed to the corresponding author.

## Author Contributions

The author confirms being the sole contributor of this work and has approved it for publication.

## Conflict of Interest

The author declares that the research was conducted in the absence of any commercial or financial relationships that could be construed as a potential conflict of interest.

## Publisher’s Note

All claims expressed in this article are solely those of the authors and do not necessarily represent those of their affiliated organizations, or those of the publisher, the editors and the reviewers. Any product that may be evaluated in this article, or claim that may be made by its manufacturer, is not guaranteed or endorsed by the publisher.
